# MAB-DrNet: Bearing Fault Diagnosis Method Based on an Improved Dilated Convolutional Neural Network

**DOI:** 10.3390/s23125532

**Published:** 2023-06-13

**Authors:** Feiqing Zhang, Zhenyu Yin, Fulong Xu, Yue Li, Guangyuan Xu

**Affiliations:** 1 Shenyang Institute of Computing Technology, Chinese Academy of Sciences, Shenyang 110168, China; 2 University of Chinese Academy of Sciences, Beijing 100049, China; 3 Liaoning Key Laboratory of Domestic Industrial Control Platform Technology on Basic Hardware and Software, Shenyang 110168, China

**Keywords:** fault diagnosis, dilated convolution, deep learning, residual network, noisy environment

## Abstract

Rolling bearing fault diagnosis is of great significance to the safe and reliable operation of manufacturing equipment. In the actual complex environment, the collected bearing signals usually contain a large amount of noises from the resonances of the environment and other components, resulting in the nonlinear characteristics of the collected data. Existing deep-learning-based solutions for bearing fault diagnosis perform poorly in classification performance under noises. To address the above problems, this paper proposes an improved dilated-convolutional-neural network-based bearing fault diagnosis method in noisy environments named MAB-DrNet. First, a basic model called the dilated residual network (DrNet) was designed based on the residual block to enlarge the model’s perceptual field to better capture the features from bearing fault signals. Then, a max-average block (MAB) module was designed to improve the feature extraction capability of the model. In addition, the global residual block (GRB) module was introduced into MAB-DrNet to further improve the performance of the proposed model, enabling the model to better handle the global information of the input data and improve the classification accuracy of the model in noisy environments. Finally, the proposed method was tested on the CWRU dataset, and the results showed that the proposed method had good noise immunity; the accuracy was 95.57% when adding Gaussian white noises with a signal-to-noise ratio of −6 dB. The proposed method was also compared with existing advanced methods to further prove its high accuracy.

## 1. Introduction

Fault diagnosis is an important part of rotating machinery equipment health management and is of great significance to the healthy and safe operation of manufacturing equipment [1]. As a core component of rotating machinery, the engine’s complex structure brings a high cost to the disassembly and assembly of the engine. Rolling bearings, as their internal core components, have a harsh working environment [2]. Once a fault occurs, it will bring huge economic losses or even casualties, so frequent testing is required [3,4]. However, frequent disassembly and assembly inspections will result in higher equipment maintenance costs. With the continuous development of information technologies, it has become a research hotspot in the field of manufacturing to deploy computer technologies to carry out non-disassembly fault diagnosis on rolling bearings of rotating machinery equipment to reduce equipment maintenance costs [5].

Fault diagnosis can be realized by analyzing the various sensing signals during engine bearing operation. The types of signals include vibration signals, acoustic emission signals and current signals, etc. [6,7,8]. The most-commonly used signal acquisition method is the acquisition of vibration signals from bearing rotation through the installation of acceleration sensors. A summary of the literature showed that intelligent fault diagnosis solutions mainly include model-based methods, empirical-based methods, traditional machine-learning-based methods, and deep-learning-based methods [9]. Among them, the model-based approach requires an accurate physical model, a large amount of a priori knowledge, and an accurate understanding of the engine system structure and parameters, while the empirical approach requires a priori knowledge for judgment and reasoning, neither of which is suitable for non-domain experts [10]. The main methods used in the domain of computing are traditional machine learning methods and deep-learning-based methods. Although traditional machine learning methods have been widely used and researched for many years [11,12,13], they have some drawbacks. First, the vibration signal extraction method based on traditional machine learning relies heavily on manual work, requiring professional experience and knowledge. The process is complex, and the workloads are heavy, which makes it extremely difficult to meet the actual needs of bearing fault diagnosis [14]. In addition, traditional machine learning methods are shallow learning with a limited ability to extract nonlinear features. As deep learning techniques are becoming more mature, their powerful feature extraction and representation capabilities offer the possibility of separating bearing state signals in complex situations.

Deep learning models commonly used in fault diagnosis include convolutional neural networks [15], recurrent neural networks [16], etc. In particular, convolutional neural network models are extensively used in fault diagnosis due to their powerful feature extraction and scalability, and the various techniques adopted by CNNs can more effectively extract the characteristics of bearing faults. Among them, one common technique is to broaden the local perceptual field of the convolutional layer, which allows the network to better capture local features and improve the accuracy of feature extraction. In addition, CNNs utilize weight-sharing techniques, where the same weight parameters are distributed to different locations, which allows the network to simplify the parameters and train faster and learn the features of bearing faults more effectively. The integration of these techniques allows the CNN to extract bearing fault features more quickly and accurately, leading to efficiency promotion for fault diagnosis. For example, Reference [17] proposed the MA1DCNN network to improve fault diagnosis accuracy by adaptively correcting the features of each layer in the network. Reference [14] proposed a novel GNR-based end-to-end CNN model to enhance network fault diagnosis. Reference [18] proposed fault diagnosis by using wide convolutional kernels. Reference [19] used normalized diagnostic feature maps and convolutional neural networks for bearing fault diagnosis. Reference [20] proposed a lightweight CNN framework that requires fewer parameters to achieve high accuracy.

Although the above methods have achieved satisfactory diagnostic accuracy, the data tested in these methods are mostly “clean” data collected in laboratories. However, at the actual site, bearings operate in harsh environments with high temperatures, high pressures, and high loads, which makes it extremely prone to fault. Therefore, regular maintenance is necessary to ensure their optimal performance. However, the signals collected by the sensors contain a large number of vibration signals from other mechanical components, and a large amount of ambient noises accumulate during the transmission process. According to the central limit theorem, we assumed that the vibration sources of each element are independent, each vibration source will generate a certain random signal, and the superposition of independent signals on each other will lead to an increase in the variance of the signal, thus tending to a Gaussian noise signal. The signal is drowned out when the Gaussian signal is strong enough, resulting in an insufficient signal-to-noise ratio [21]. The above features make it more challenging to extract bearing vibration signals and reduce the accuracy of bearing fault diagnosis.

To improve the noise immunity of convolutional neural networks, different CNN-model-based denoising methods have also been proposed by researchers. A method based on wavelet transform (WT) and an improved residual neural network was proposed by [9], but its use of SVD to improve the pooling layer made it less efficient. Reference [22] proposed combining PCA theory with deep learning technology and applying it to a high-speed rail system for early fault diagnosis.Reference [23] proposed a deep learning method for bearing fault diagnosis by superimposing residual null convolution, but it only considered a single load situation and a high signal-to-noise ratio, which cannot meet the requirements of practical complex environments. Reference [24] proposed a model with high fault diagnosis accuracy based on a working mechanism of soft thresholding and global context, but its sharing of a threshold value for all channels led to ignoring the possibility of different amounts of noise features in different channels.Reference [25] proposed a deep transient feature learning method that forms a training dataset by simulating the underlying signals of different pulse wavelet bases and learns to anticipate repetitive transient pulse features during the training process, which in turn constructs a mapping of noisy TFDs to clean TFDs; however, the method is only used to remove noise, and there is a lack of fault diagnosis. Reference [26] presented a hierarchical-branch-based bearing fault diagnosis method using convolutional neural networks (CNNs) and conducted experiments in the SNR range of 2 dB to 12 dB, achieving good results. Reference [27] proposed a bearing fault diagnosis method based on multi-granularity information fusion, which enhanced robustness against noise. However, the experiments regarding the noise environment were relatively simple. Reference [28] introduced a bearing fault diagnosis method under noisy backgrounds using adaptive thresholding, achieving high diagnostic accuracy; however, the model complexity was high, and the designed experiments did not include comparisons under low signal-to-noise ratios. Reference [29] proposed a novel error cost function and a structure adaptive algorithm based on stacked denoising auto-encoders. However, it only verified noise accuracy at higher signal-to-noise ratios.

Therefore, it remains a major challenge to realize a bearing fault diagnosis method under noisy conditions. Due to the presence of noise, fault diagnosis can suffer from signal distortion, decreased signal-to-noise ratios, and an increased risk of misdiagnosis. Therefore, effective noise suppression and feature enhancement strategies are necessary for accurate fault diagnosis. However, existing noise suppression and feature enhancement methods face limitations in addressing the impact of noise on fault diagnosis. These limitations include limited noise suppression effectiveness and potential feature loss. To address the above problems, this paper proposes a bearing fault diagnosis method based on an improved dilated convolutional neural network to achieve feature extraction and fault diagnosis of bearing vibration signals in noisy environments. The proposed method is consistent with the research trend in the field of bearing fault diagnosis, and has both theoretical significance and application value. In this paper, we designed a bearing fault diagnosis method consisting of a short-time Fourier transform and MAB-DrNet. The original signal was converted into a time–frequency map by the short-time Fourier transform, and then, the proposed MAB-DrNet was used for feature extraction and bearing fault diagnosis. The main contributions of this paper are as follows:

1. An innovative bearing fault diagnosis model, MAB-DrNet, was designed for accurately determining the health status of bearings in noisy environments.
2. A residual network based on dilated convolution was designed, which increased the perceptual field of the convolution kernel and improved the accuracy of bearing fault diagnosis by using dilated convolution instead of conventional convolution.
3. We propose the MAB module, which selectively focused on informative features, allowing the model to allocate its resources more effectively and enhance its ability to distinguish relevant and irrelevant features. This improved the overall performance of the model. Additionally, we introduced the global residual block to address the issues of vanishing and exploding gradients, thereby improving the network’s generalization capability and robustness.
4. The experimental validation was carried out on a bearing vibration dataset using the Case Western Reserve University dataset. The proposed method was validated and compared by adding different intensities of noise to the dataset, and the effectiveness of the proposed method is demonstrated.

The remainder of this paper is organized as follows. Section 2 reviews the short-time Fourier transform, dilated convolution, and residual network and analyzes their advantages. Section 3 introduces the proposed fault diagnosis method. Section 4 validates the performance of the proposed model by using the CWRU dataset for comparative analysis with several models and illustrates the necessity of the proposed modules through ablation experiments. Section 5 concludes the paper and discusses future work.

## 2. Methodology

This section introduces the related research work. First, the advantages and principles of the short-time Fourier transform in non-stationary signal processing are introduced, and its parameter settings in this paper are presented. This is followed by a detailed description of the principles of dilated convolution. Finally, the characteristics of the residual network and the principle are discussed.

### 2.1. Short-Time Fourier Transform

**Figure 1:**
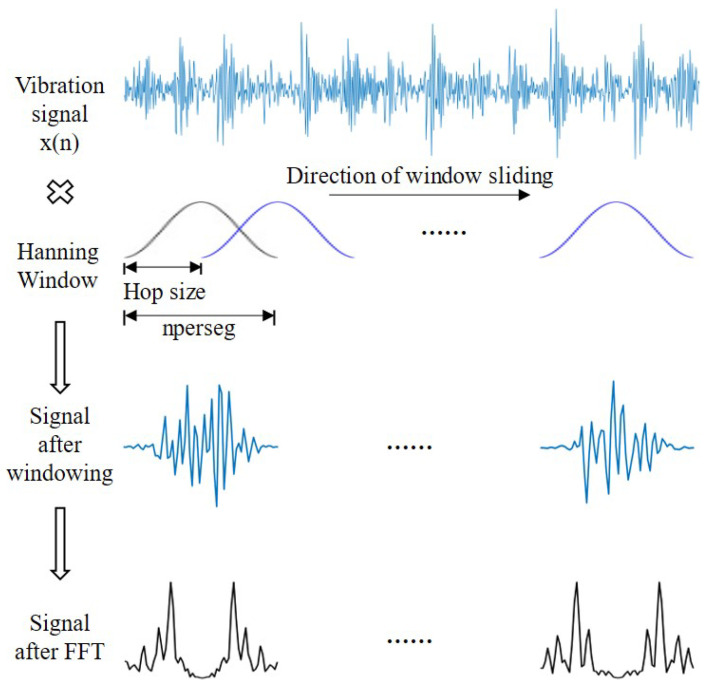
The implementation process of the short-time Fourier transform.

Short-time Fourier transform and wavelet transform are commonly used methods in signal processing, both of which can provide frequency domain and time domain information of signals and are methods for analyzing non-stationary signals. Wavelet transform is more suitable for dealing with local features of non-stationary signals, but its results are affected by the selection of wavelet bases, which requires a certain amount of experience [30]. The STFT is relatively easy to explain and implement. Therefore, the short-time Fourier transform is a more suitable signal analysis method for non-stationary signals such as bearing vibration signals. The process of the short-time Fourier transform is to divide the signals into multiple time periods so that the signals in each time period can be regarded as stationary signals, and then, the signals in each time period can be Fourier transformed to obtain the frequency domain information in each time period, so that the frequency domain information and time domain information of the signals can be obtained. The process of the short-time Fourier transform is shown in Figure 1.

As shown in Figure 1, the signals were first divided into multiple time segments. To reduce the fluctuation of spectrum estimation and reduce the impact of spectral leakage, overlapping is usually performed. This involves some overlap between adjacent time periods. In this paper, the length of the signals in each period was set to 64, and there was an overlap between the time segments, which was set to 50%. To reduce the discontinuity of the signals at the endpoints of the time segments, we used the Hanning window to add windows to the signals within each time segment. The Hanning window is a commonly used window function with the formula:

(1) $$w\left(n\right)=0.5\left(1+cos\left(2\pi \frac{n}{M-1}\right)\right),$$

where *m* represents the effective length of the window function, *n* represents the sample number in the current window, and n=0,1,2,…,M−1.

Then, apply a selected window function to different time segments of the original signals, usually by shifting the position of the window to cover the entire signal. The signals in each time period are multiplied with the window function, and the equation is as follows:

(2) $$y\left(n_{1}\right)=x\left(n_{1}\right)w\left(n_{1}-t\right).$$

Next, the Fourier transform is applied to the signals within each time period to obtain the frequency domain information within each time period.

Finally, the frequency domain information within each time period is stitched together to obtain the time–frequency information of all the signals. The STFT formula is shown in Equation (3):

(3) $$X\left(m,\omega \right)=\sum_{n=0}^{n=M-1}x\left(n\right)w\left(n-t\right)e^{-j\omega n},$$

where *t* represents the index of the time period, x(n) represents the input signals, w(.) represents the window function, and ω represents the frequency.

Through these steps, the STFT divides the original signal into multiple time segments by windowing, applies a window function to the signal in each time segment, and finally, obtains the frequency domain representation of each time segment. In this way, the change information of the signal at different times and frequencies can be obtained, which was used to analyze the spectral characteristics of the time-varying signal.

### 2.2. Dilated Convolution

To improve the accuracy of convolutional neural networks in processing tasks, researchers usually increase the depth of the network, that is increase the convolutional layer and pooling layer of the network. However, increasing the network depth will also lead to an increase in the number of parameters and hyperparameters, which consumes considerable computing resources and storage resources of the network. In addition, due to the excessive number of network layers, deep networks are prone to the problems of gradient disappearance and gradient explosion, resulting in a sharp decline in network performance, and sometimes even saturation. Therefore, when designing a deep model, it is necessary to consider how to solve these problems to achieve a better performance and effect.

Dilated convolution was first proposed by Fisher Yu et al. [31]. This convolution operation expands the receptive field of the convolution kernel by inserting holes in the convolution kernel, thereby increasing the effective receptive field of the convolution layer without increasing the number of parameters so that the depth model can better process large-scale images and speech signals efficiently. Therefore, dilated convolution has achieved excellent performance in many vision and speech tasks [32,33] and has become an important part of modern deep learning.

We know that the number of layers, stride, and padding size of the network model will affect the receptive field size of the convolution kernel of the convolutional layer. In order to explain the principle of dilated convolution more clearly, we start from the traditional convolution and introduce how to calculate the size of the convolution kernel receptive field. Generally, we can use Equation (4) to calculate the output size of the lth convolutional layer as follows:

(4) $$n_{l}=\left\lfloor \frac{n_{l-1}-f+2p}{s}\right\rfloor +1,$$

where $n_{l-1}$ indicates that the previous layer is the output size, that is the input size of this layer; $n_{l}$ indicates the output size of this layer; *f* indicates the size of the convolution kernel; *p* indicates the filling size; and *s* indicates the stride. Through the above formula, we can calculate the output size of each layer of convolution.

The dilated convolution neural network uses dilated convolution instead of ordinary convolution, and ordinary convolution can also be regarded as a special case of dilated convolution. We can obtain the output size when using dilated convolution by adding the dilation rate *d* to Equation (4), as shown in Equation (5):

(5) $$n_{l}=\left\lfloor \frac{n_{l-1}-f-d+2p}{s}\right\rfloor +1.$$

We assumed that the receptive field size of the *l*th convolutional layer is $r_{l}$; then,

(6) $$r_{l}=r_{l-1}+d_{l}*\left(f_{l}-1\right)*\prod_{i=1}^{l-1}s_{i},$$

Among them, the initial value of the receptive field $r_{0}=1$. We set the stride of hole convolution to s=1. The reason we set the stride of the dilated convolution s=1 was that when the stride is 1, the distance between pixels remains 1 as the convolution kernel moves over the feature map and no holes appear, thus maintaining the validity and stability of the convolution. If the stride of the dilated convolution is set to a value greater than 1, then as the convolution kernel moves on the feature map, the distance between pixels will become larger and holes will appear, causing the convolution operation to no longer be effective. In addition, if the stride is too large, the output size will shrink too quickly, which will affect the coverage and receptive field size of the network, thereby affecting the performance and feature extraction ability of the network. Therefore, when using hole convolution, the stride is usually set to 1 to ensure the effectiveness and stability of the convolution operation, so Equation (6) is simplified to Equation (7).

(7) $$r_{l}=r_{l-1}+d_{l}*\left(f_{l}-1\right).$$

**Figure 2:**
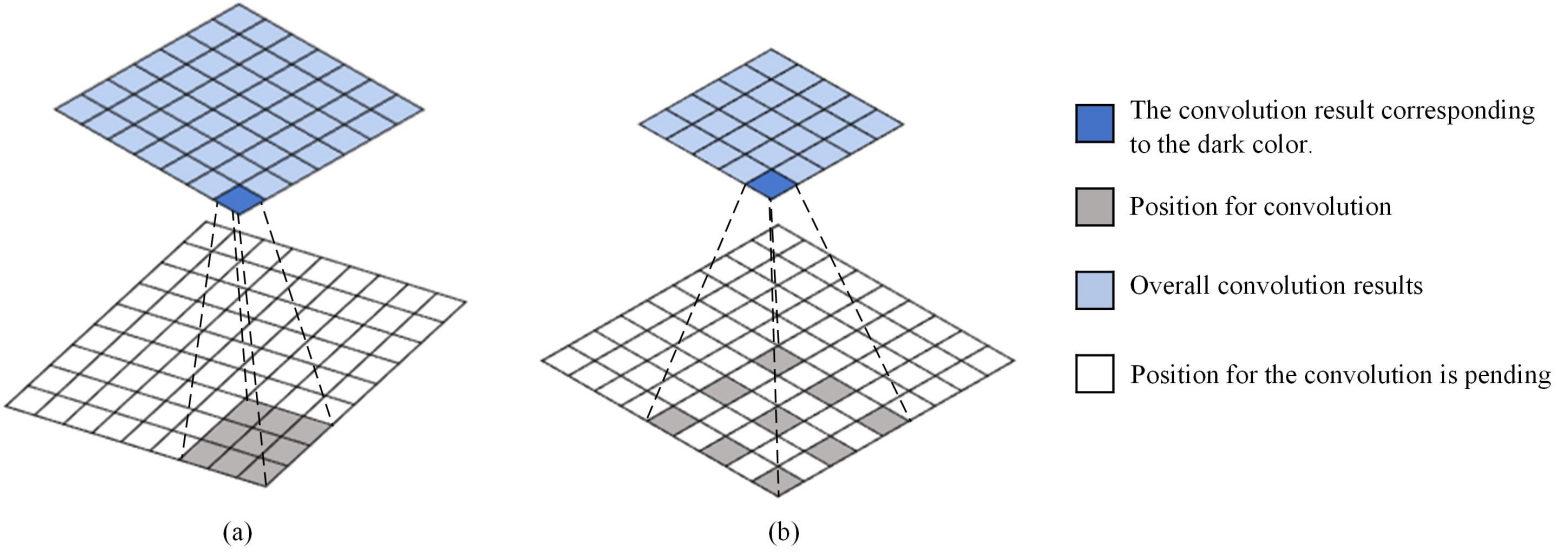
Schematic diagram of the receptive field during the dilated convolution expansion process, where (**a**) represents the ordinary convolution schematic and (**b**) represents the dilated convolution schematic.

To understand the implementation process of dilated convolution more intuitively, the receptive field diagram of dilated convolution under different dilated rates is shown in Figure 2. Among them, in Figure 2a, the dilation rate was set to 1, and its implementation process was consistent with ordinary convolution; in Figure 2b, the dilation rate was set to 2.

As mentioned above, the introduction of dilated convolution can increase the receptive field. This paper studied the technology based on the introduction of dilated convolution to increase the receptive field, but it is necessary to choose an appropriate receptive field size when using this technology because if the receptive field is too small, the network cannot capture the global information in the image, resulting in network performance decline. In contrast, if the receptive field is too large, you need to increase the padding to ensure that the output size remains unchanged, which will lead to a decrease in network performance and will significantly increase the number of calculations and parameters of the network, resulting in increased training time and consumption of computing resources, as well as being prone to overfitting. Therefore, to achieve the ideal model effect, we conduct the void rate selection experiment in Section 4.

### 2.3. Residual Model

**Figure 3:**
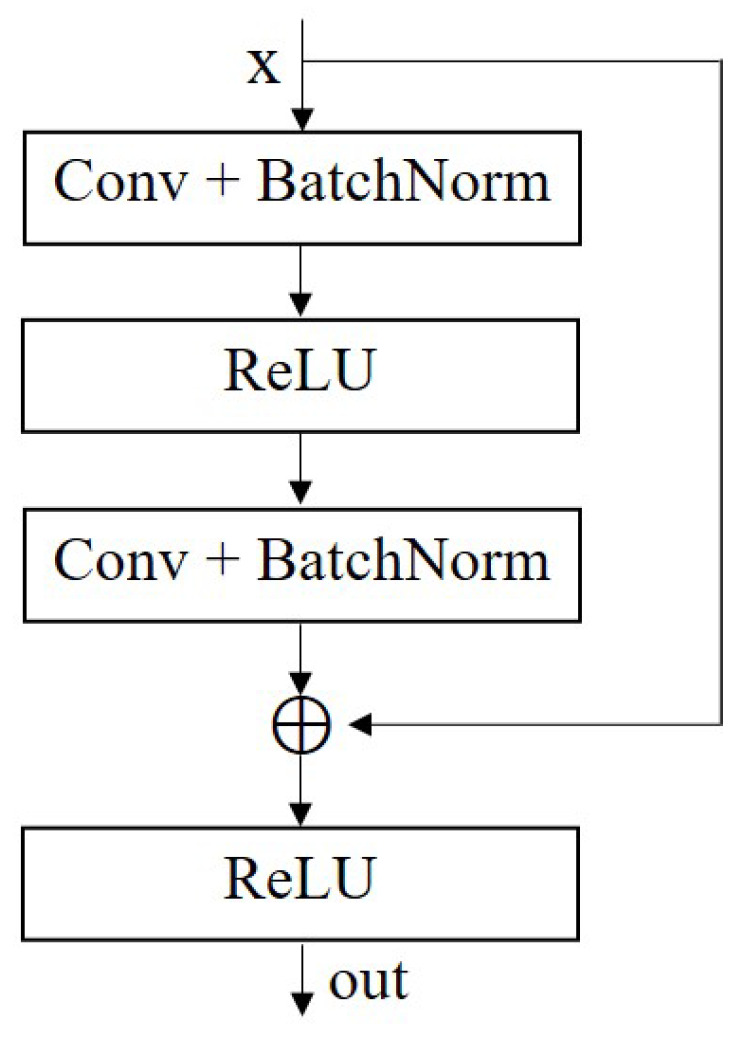
Diagram of the residual block.

A residual network is a deep convolutional neural network that enables the network to learn complex features more easily by using residual modules to learn cross-layer mappings. The problems of vanishing and exploding gradients are avoided [34]. The basic idea of the residual block is that, in the process of transmitting information, the network can skip some layers and directly pass the input to the subsequent layer, keeping the original information unchanged and performing subsequent processing, thus avoiding the loss or disappearance of information. This method allows the residual network to be trained at a deeper level, improving the performance and effectiveness of the network. The schematic diagram of the residual module is shown in Figure 3, and its definition is as follows:

(8) out=ReLU(x+F(x)),

where *x* represents the input data, f(.) represents the output result after a series of convolution, BatchNorm, and ReLU operations, and out represents the output of this module.

## 3. The Proposed Method

This section proposes a new bearing fault diagnosis model, MAB-DrNet. First, the overall structure of the proposed model is introduced. Then, the structure and roles of its constituent components, DrNet, MAB, and GRB, are introduced.

### 3.1. The Proposed Bearing Fault Diagnosis Framework

**Figure 4:**
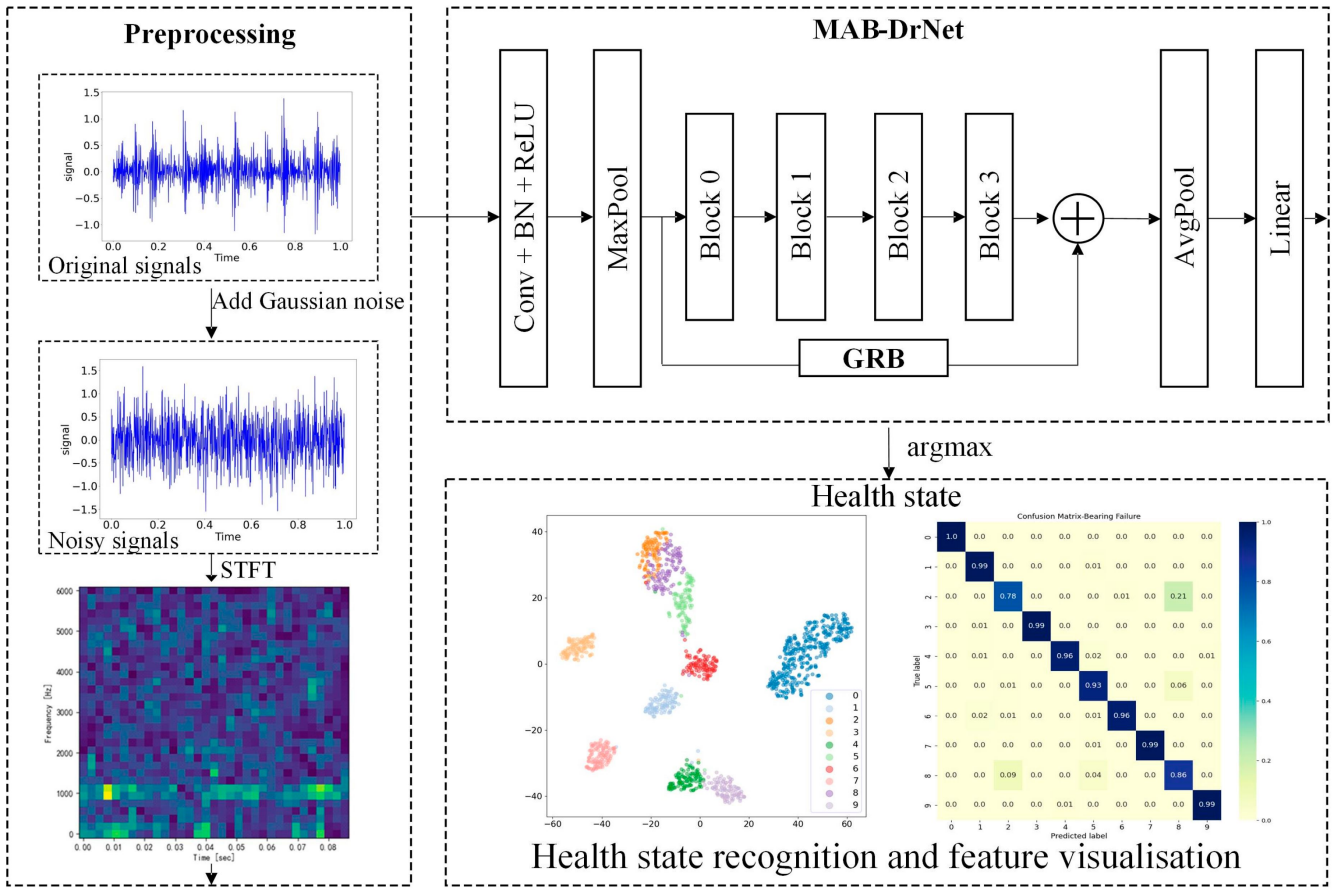
The framework of the proposed method for fault diagnosis.

In practical applications, the collected vibration signals are usually inevitably superimposed with a large amount of noises, which will lead to the deterioration of the feature extraction and learning ability of the convolutional neural network, thereby affecting the accuracy of bearing fault diagnosis. To solve this problem, we propose a bearing fault diagnosis method based on a short-time Fourier transform and MAB-DrNet, and the overall architecture diagram is shown in Figure 4. The proposed approach comprises two main components: data preprocessing and the classification model MAB-DrNet. In the data preprocessing stage, we employed an acceleration sensor to collect vibration signals, which were then transformed into a time–frequency representation known as the time–frequency diagram. This conversion technique enabled us to represent the one-dimensional time-series signals in a two-dimensional format that effectively captures the signal characteristics. By analyzing the signals in both the time and frequency domains, we can more accurately extract fault-related features, thus enhancing the diagnostic accuracy. The classification model, MAB-DrNet, played a crucial role in our approach. It leverages the benefits of attention mechanisms and deep residual networks to effectively capture complex patterns and correlations within the transformed signals. The MAB-DrNet model was designed to learn discriminative representations from the time–frequency diagrams and perform accurate classification of various fault types.

**Figure 5:**
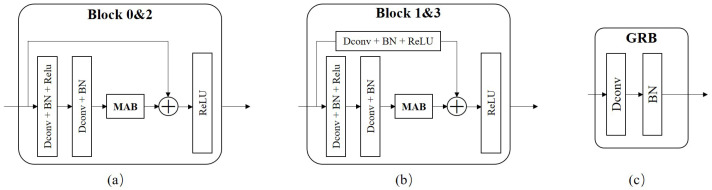
The dilated convolutional network in the proposed method includes: (**a**) Block 0 and Block 2 modules; (**b**) Blocks 1 and 3; (**c**) global residual block.

The MAB-DrNet model we designed is a classification model for bearing fault diagnosis. We first propose a basic model based on the dilated convolutional neural network, which uses the technology of dilated convolution and residual connection to improve the effect of feature extraction. Based on this, we propose the MAB-DrNet model. The MAB-DrNet model is mainly composed of four blocks and a GRB block. Among them, Block 0 and Block 2 have the same structure, as shown in Figure 5a; Block 1 and Block 3 have the same structure, as shown in Figure 5b. It can be seen from the figure that the input data pass through two convolutional layers, two batch normalization layers, and two activation layers, and to further improve the accuracy, we introduced the MAB we designed before the last activation function module, which can improve the network’s attention to important features. At the same time, MAB-DrNet also includes the GRB block, which is able to learn features globally to improve diagnostic accuracy. In the implementation phase, we input the time–frequency map into the MAB-DrNet model for fault classification. The implementation of the algorithm is shown in Algorithm 1.
**Algorithm 1** Implementation process of the proposed fault diagnosis model**Input:** dataset *X* and their labels**Output:** bearing health state  1: Normalize the data in the dataset *X* to [0,1]: $X_{normalize}=Normalization\left(X\right)$  2: Conduct time–frequency maps: $X_{STFT}=STFT\left(X_{normalize}\right)$  3: Orderly divide the dataset into training sets $X_{train}$ and test sets $X_{test}$, and $X_{train}:X_{test}=8:2$  4: Initialize the model with parameters θ.  5: **for** epoch in 0 to epochs - 1 **do**  6:       Calculate the output value of the model logits, $logits=Model(X_{train})$.  7:       Calculate the loss, loss=CrossEntropyLoss(logits,labels).  8:       Update parameters θ using back propagation.  9: Calculate the predict value, $predict=Model(X_{test})$10: **return** the bearing heath state

### 3.2. The Proposed Dilated Residual Network

A dilated residual network is a deep convolutional neural network model based on residual blocks. Its basic block is composed of a dilated convolution layer (Dilated Conv), a batch normalization layer (BatchNorm), and an activation function (ReLU). These three layers work together on the data, which can make the model better extract features, normalize the data distribution, and enhance the nonlinear expression ability of the model, thereby improving the performance of the model. Among them, the batch normalization layer can accelerate the convergence speed of the network, suppress gradient disappearance or explosion, and increase the generalization ability of the network. A dilated convolutional layer is a convolutional layer with an adjustable receptive field, which can perform comprehensive convolution on the image to extract richer information from the input image. When using dilated convolution, the dilation rate is 1, meaning that the convolution layer is a conventional convolution. When the dilation rate is greater than 1, this means that there is an interval between the elements inside the convolution kernel, thereby increasing the receptive field of the convolution kernel. In this way, using dilated convolution can effectively expand the receptive field of each layer to improve the model’s ability to extract larger-scale features in the image. When the dilation rate is greater than 1, to ensure that the output size remains unchanged, padding needs to be used, and stride = 1 was set at the same time. We chose 3 × 3 as the size of the convolution kernel. Table 1 shows the structural parameter configuration of the DrNet model, and the dilation rate was set to 1, 7, and 1, respectively. The selection verification experiments of the dilation rate are presented in Section 4.

### 3.3. The Introduced Global Residual Block

The global residual block (GRB) was introduced to address the problem of information loss associated with operations such as convolution. In deep neural networks, information declines as the number of layers increases, which can lead to performance degradation. The global residual block solves this problem by introducing cross-layer connections. Specifically, the global residual module adds up the input and output data in order to pass the residual information to the next layer, thus preserving the information of the input data while better passing on the gradient information, making the neural network easier to optimize and train. This cross-layer connection can effectively reduce information loss and improve the performance and accuracy of the network. The structure is shown in Figure 5c.

As seen from the figure, our GRB block consisted of a convolutional layer and a batch normalization layer. Here, the convolutional layer uses ordinary convolution, that is the dilation rate was set to 1. This is because, in this case, compared with the dilated convolution, the ordinary convolution has fewer calculations and parameters, so it can improve the calculation efficiency and accuracy of the model to a certain extent, without significantly affecting the performance of the model. Therefore, choosing to use ordinary convolution instead of dilated convolution in this block can better balance the performance and complexity of the model.

### 3.4. The Designed MAB Module

**Figure 6:**
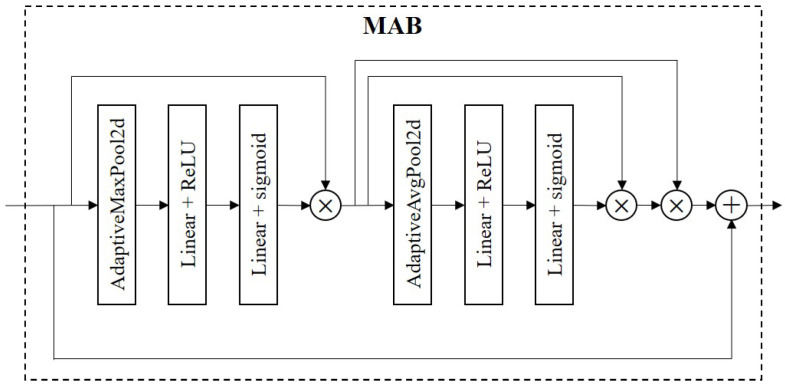
The structure of MAB.

The structure of the MAB block is shown in Figure 6. The design of our proposed MAB block drew on the ideas of the squeeze-and-excitation (SE) attention ResNet [35] developed for improving the performance of image recognition tasks. The SE module enhances the feature channels with important information by learning the importance of each feature channel, thus improving the performance of the model. The MAB block builds on the SE block and is used to find a balance between computational efficiency and accuracy.

The main structure of the MAB block includes pooling layers, fully connected layers, and activation function layers. Specifically, in the MAB block, we first used the maximum pooling layer to compress the information of each channel to reduce the computational burden and improve the computational efficiency of the model. Next, the pooled feature maps are fed into the fully connected layer for feature transformation, the ReLU activation function is used for nonlinear transformation, and the input value is mapped to a non-negative range, thereby increasing the nonlinear capability of the model and helping improve the performance of the model. Moreover, the module passes through a fully connected layer and a sigmoid activation function to map the input value to the interval between 0 and 1, indicating the importance of each feature channel, reducing the risk of overfitting and increasing the generalization ability. To avoid the vanishing gradient problem and speed up training, this module introduces a residual connection, which adds the output of the previous layer to the output of the current layer. The above process can be expressed by Equation (9):

(9) $$y_{1}=\sigma \left(FC\left(\delta \left(FC\left(MaxPool\left(x\right)\right)\right)\right)\right)⊗x,$$

Among them, MaxPool represents the adaptive maximum pooling layer, FC represents the fully connected layer, δ represents the activation function ReLU, and σ represents the activation function sigmoid.

(10) $$y_{se}=\sigma \left(FC\left(\delta \left(FC\left(AvgPool\left(y_{1}\right)\right)\right)\right)\right)⊗y_{1}.$$

(11) $$y_{1}=y_{se}\left(y_{1}\right)⊗y_{1}.$$

(12) $$y=y_{2}⊕x.$$

The resulting output is then fed into the SE module to obtain the importance of each feature channel, and these importance levels are used to assign a weight value to each feature channel, which is then multiplied directly with the output of the previous layer, as shown in Equations (10) and (11). Finally, these weight values are weighted and summed with the output of each channel in the previous layer of the feature map to obtain the final output, as shown in Equation (12).

Through these steps, the MAB module can adaptively adjust the weight of each channel according to the importance of each channel to improve the network’s ability to identify key features, thereby improving the performance and accuracy of the entire model.

## 4. Experimental Verification and Analysis

In this section, we first give the setting of parameters of the experiment and the evaluation metrics, and then, we present the dataset. Then, an experimental comparison and analysis of the proposed model were carried out, and the validity and rationality of the proposed model were verified and analyzed through ablation experiments.

### 4.1. Experimental Setup and Evaluation Metrics

#### 4.1.1. Experimental Settings

The implementation of the model and the corresponding experimental verification were carried out on a high-performance computer with an Nvidia GeForce RTX 2080Ti GPU using the PyTorch 1.11 framework. The optimizer uses Adam; the batch size was set to 32; the initial value of the learning rate was set to 0.001; the decay mode of the learning rate was set to step and decayed every 10 epochs; the dropout was set to 0.02; the CrossEntropyLoss was used as the loss function. The number of iteration epochs was set to 50. We set a specific random seed to ensure that the random process involved in the experiment produced consistent results across runs.

#### 4.1.2. Evaluation Metrics for Classification Performance

The MAB-DrNet model we proposed is a classification model. We used the accuracy rate to judge the effect of the fault diagnosis model, which is shown in Equation (13):

(13) $$Accuracy=\frac{TN+TP}{TN+TP+FP+FN},$$

Among them, TP stands for true positive, TN stands for true negative, FP stands for fault positive, and FN stands for fault negative.

### 4.2. Dataset Description

**Figure 7:**
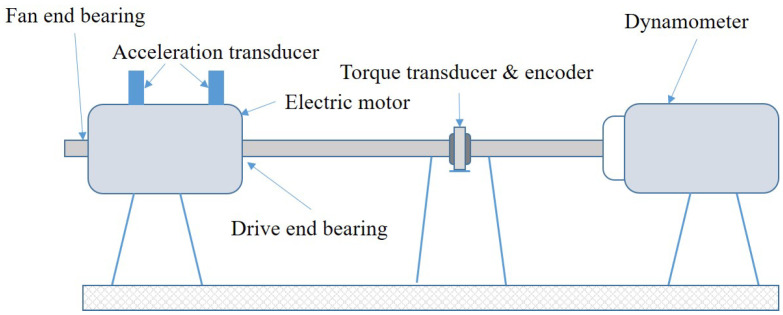
CWRU data acquisition device.

The Case Western Reserve University (CWRU) dataset [36] was chosen as the data for our experiments to validate the effectiveness of the proposed model. The data acquisition device consisted mainly of an electric motor, a torque transducer, and a dynamometers, as shown in Figure 7. With a motor horsepower of 2HP, the single-point fault was placed on the bearing (SKF6205) by EDM machining techniques, and the original vibration signals were obtained by the acceleration transducer laid on the bearing. The bearing state types included four states: normal state, inner ring fault, outer ring fault, and rolling element fault located between the inner and outer rings. Each fault type was classified as 0.007 inches, 0.014 inches, and 0.021 inches by artificial processing damage. Ten bearing conditions were selected in this paper, as shown in Table 2. We chose the drive end accelerometer data with a sampling frequency of 12 kHz and mixed the bearing data of four loads of 0 hp (1797 rps), 1 hp (1772 rps), 2 hp (1750 rps), and 3 hp (1730 rps) as the object of our experiment, to increase the diversity of data and improve the generalization ability of the model. Among them, we set the length of each signal sample to 1024 for non-overlapping sampling. We divided 80% of the samples in order as the training dataset and 20% of the signal samples as the test dataset.

### 4.3. Data Preprocessing Method Based on STFT

Generally, the engine bearing signal acquisition test bench built in the laboratory can reflect the fault characteristics of the bearings in the real environment, but because the laboratory environment is more ideal, although there is inherent noise in the vibration signals obtained from the test bench, the coupling between the equipment is high and the complexity is relatively low, so the bearing signals collected in the test bench may be too ideal. Compared with the real environment, there is a certain gap, which cannot fully reflect the real and complex on-site working conditions. To verify the validity of the model under various background noises, scholars generally add Gaussian white noise of different strengths to the collected signals.

Therefore, similarly, we evaluated the performance of our proposed MAB-DrNet model by adding Gaussian white noise to the gathered vibration signals to restore the actual environment as much as possible. We evaluated the strength of the noises by the signal-to-noise ratio, which is defined as shown in Formula (14):

(14) $$SNR=10\log_{10}\left(\frac{x}{n}\right),$$

where *x* represents the signal power and *n* represents the noise power in dB.

**Figure 8:**
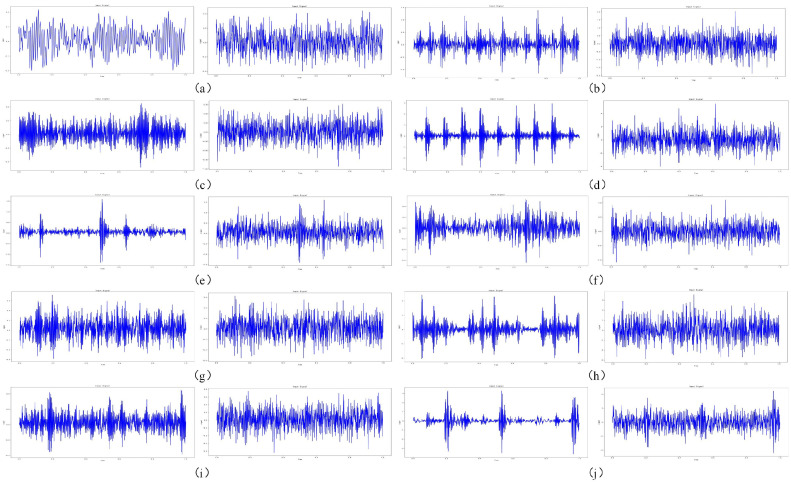
The time domain image representation of raw signals and the addition of a white Gaussian noise signal with an SNR of −4 dB of (**a**) normal, (**b**) 0.007 in inner fault, (**c**) 0.007 in Ball fault, (**d**) 0.007 in outer fault, (**e**) 0.014 in inner fault, (**f**) 0.014 in ball fault, (**g**) 0.014 in outer fault, (**h**) 0.007 in inner fault, (**i**) 0.007 in ball fault, and (**j**) 0.007 in outer fault.

**Figure 9:**
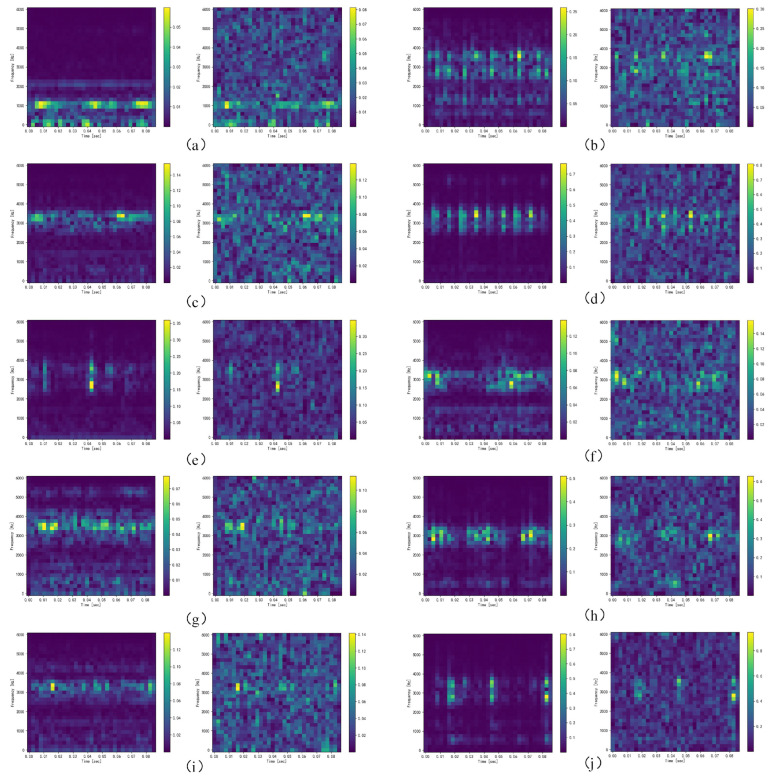
Time–frequency image representation of raw signals and the addition of a white Gaussian noise signal with an SNR of −4 dB of (**a**) normal, (**b**) 0.007 in inner fault, (**c**) 0.007 in ball fault, (**d**) 0.007 in outer fault, (**e**) 0.014 in inner fault, (**f**) 0.014 in ball fault, (**g**) 0.014 in outer fault, (**h**) 0.007 in inner fault, (**i**) 0.007 in ball fault, and (**j**) 0.007 in outer fault.

Figure 8 shows the comparison of the time domain diagrams of 10 kinds of bearing health statuses before and after adding Gaussian white noise with a signal-to-noise ratio of −4 dB to the original signals, where the horizontal axis represents the sampling time and the vertical axis represents the amplitude. It can be seen from the figure that the characteristics of the signals were submerged after adding noise, which will greatly increase the difficulty of fault diagnosis. Therefore, we converted the bearing vibration signals with noise into a time–frequency diagram through a short-time Fourier transform and analyzed the signals of different frequency bands through the combination of frequency domain decomposition and time domain decomposition, so that it is easier to find fault modes and features. At the same time, the short-time Fourier transform has an anti-interference ability and can perform localized analysis on the signals in the two dimensions of the time domain and frequency domain, reduce the influence of noise, and improve the accuracy and reliability of fault diagnosis. Therefore, the time–frequency diagram obtained by the short-time Fourier transform can analyze and diagnose the bearing vibration signals more comprehensively, eliminate or reduce the influence of noise interference, and improve the accuracy and efficiency of fault diagnosis. Figure 9 shows the time–frequency diagram of the tested bearing under the condition of Gaussian white noise with a signal-to-noise ratio of −4 dB. Before training and testing, the pixel values of all images were fixed between [0, 1], so in the experiment, the time–frequency diagram is a grey-scale diagram, and here, the RGB image is shown for clear observation.

### 4.4. Experimental Results and Comparative Analysis

When we compare with other models, we ensure that: (1) the same dataset was used for evaluation; (2) the data used were obtained from the same collection point, and the load used was also the same; (3) the sampling frequency was the same in the experiment. Only when the above conditions were met, we compared and analyzed the effectiveness of the models through the accuracy rate to ensure the fairness and reliability of the comparison.

#### 4.4.1. Comparison and Analysis of the Proposed Models

First, to verify the effectiveness of our proposed MAB-DrNet model under various background noises, we conducted the following comparative experiments, and the experimental results are shown in Table 3. Comparing the model proposed in this paper with the classic ResNet18 and EfficientNet v2, it can be seen from the table that the accuracy of our proposed model can reach the same or even higher accuracy than the above-mentioned classic model. For example, for the added noise, when it was −6 dB, the accuracy of our model was 1.17% higher than that of ResNet18 and EfficientNet v2, but our parameter amount was only approximately 1/5 and 1/2 of theirs.

We can conclude that the accuracy of our model was 95.57%, 97.91%, 98.66%, and 99.33% for SNRs of −6 dB, −4 dB, −2 dB, and 0 dB, respectively, which are better than most of the well-performing methods. This was due to the fact that the collected signal data were firstly processed from both time and frequency domains in this paper, which initially solved the problem of non-smooth and nonlinear signals. Then, the designed model incorporated a combination of residual networks and dilated convolutions to enhance its feature extraction capabilities. The residual networks, known for their ability to alleviate the vanishing gradient problem, allowed the model to effectively capture and propagate important features throughout the network. The inclusion of dilated convolutions further enhanced the receptive field of the model, enabling it to capture both local and global contextual information. To enhance the model’s attention to important features, a novel attention module called MAB was proposed in this study. The MAB selectively focused on informative features and enabled the model to allocate its resources more effectively, enhancing its ability to discriminate between relevant and irrelevant features, thereby improving the overall performance of the model. Furthermore, to address the challenge of information loss during the feature extraction process, the model incorporated global residual blocks. These blocks allowed the model to preserve essential information by providing shortcuts for the flow of information across different layers. By integrating these global residual blocks, the model ensured that important features were retained and propagated through the network, mitigating the issue of information loss and enhancing the overall representation capabilities of the model. In summary, the combination of residual networks and dilated convolutions, along with the proposed attention module (MAB) and global residual blocks, collectively enhanced the feature extraction abilities of the model. This comprehensive approach enabled the model to effectively capture relevant features, allocate attention to important information, and mitigate information loss, thereby improving its performance in the task of bearing fault diagnosis.

**Figure 10:**
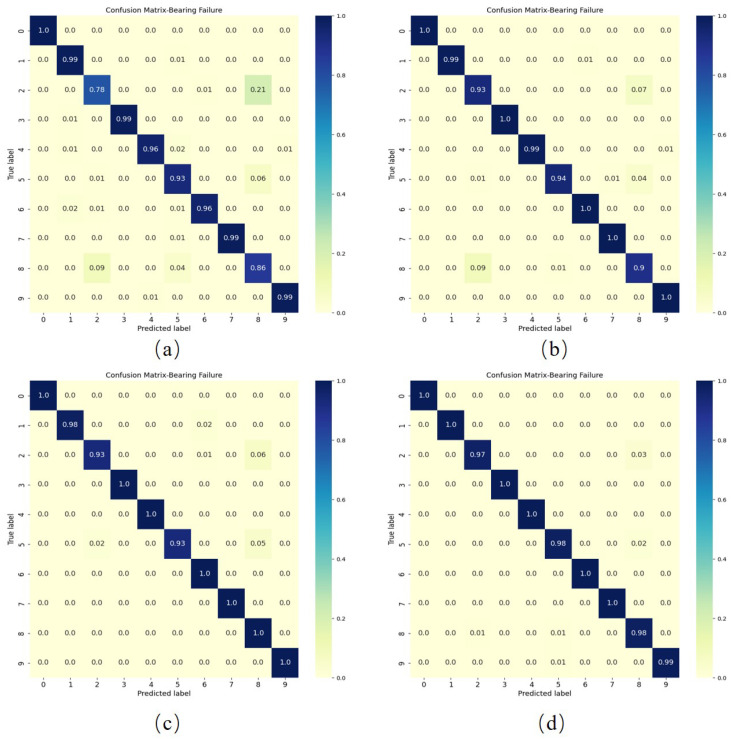
Confusion matrix for the proposed algorithm, where: (**a**) −6 dB; (**b**) −4 dB; (**c**) −2 dB; (**d**) 0 dB.

Next, we constructed a confusion matrix, as shown in Figure 10; we can intuitively reflect the classification performance of the model on different categories of data by constructing the confusion matrix, which can help us evaluate the classification effect of the algorithm more clearly. Specifically, the confusion matrix shows the correspondence between the predicted results of the classifier on each category and the actual labels, such as true positives, false positives, true negatives, false negatives, etc. For example, at 0 dB, Fault Type 2 was mistakenly classified as Fault Type 8, Fault Type 5 mistakenly classified as Fault Type 8, etc., indicating that the diagnosis of these two types was still difficult.

**Figure 11:**
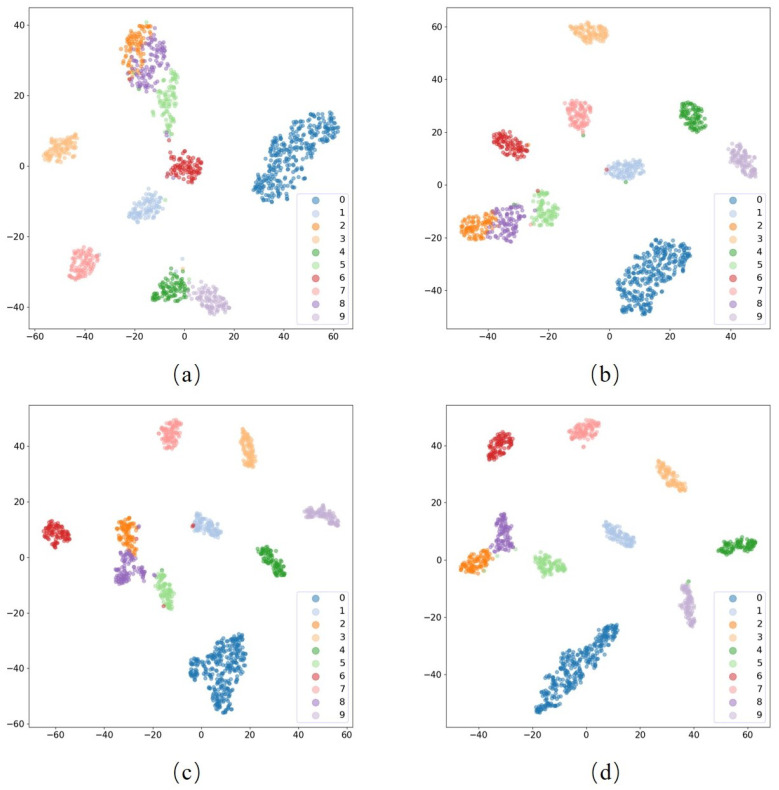
Visualization of the t-SNE of the proposed algorithm, where: (**a**) −6 dB; (**b**) −4 dB; (**c**) −2 dB; (**d**) 0 dB.

In addition, we also adopted t-distributed stochastic neighbor embedding (t-SNE) technology to map the high-dimensional features of the model into two-dimensional features while preserving the relative distance relationships between the original data, thus allowing us to better observe and understand the structure and distribution of the data to demonstrate that the proposed method can automatically learn the features of vibration signals, as shown in Figure 11. The t-SNE visualization allowed us to visualize the distribution of the data points in the 2D feature space for different categories, as well as the differences and similarities between the different categories. By analyzing the visualization results, we verified the effectiveness and reliability of the proposed algorithm.

#### 4.4.2. Ablation Experiment

1. The choice of the dilation rate:
  As mentioned in Section 2.2, the dilation rate will directly affect the denoising performance of the model, so to select an appropriate dilation rate, we chose to set the values of d to 3, 5, 7, and 9, respectively. Then, we accomplished experiments under the noise-adding condition of SNR = −4 dB, and the experimental results are shown in Table 4.
  It can be seen from the table that, as the dilation rate increased, the accuracy of the model also increased, but when the dilation rate was too large, the accuracy of the model decreased. When the dilation rate was seven, the model performed best. Therefore, the dilation rate was set to seven in our experiment.
2. The validity verification of each module of the proposed model:
  As seen from Section 3, our model consisted of three main components, including the MAB block, the GRB block, and the DrNet block, of which DrNet is the baseline model we designed. To verify the effectiveness of the block of our proposed model, we conducted ablation experiments, as shown in Table 5. Based on the experimental results, we can conclude that our proposed MAB block and GRB block both improved the accuracy of the benchmark model, and the model accuracy was also improved by adding both to our benchmark model at the same time. At the same time, we also found that, after adding the MAB and GRB modules to the benchmark model, the noise immunity of the model to high noise significantly increased. At a signal-to-noise ratio of −6 dB, the accuracy of the model increased by 1.42%.

## 5. Conclusions

To solve the problem of noise leading to the non-smooth and nonlinear issues of the collected signal wave affecting the accuracy of bearing fault diagnosis, a rolling bearing fault diagnosis method based on an improved dilated convolutional neural network, namely MAB-DrNet, was proposed. The method integrated a short-time Fourier transform and improved dilated convolution. First, we proposed an STFT-based data preprocessing method by which the time-domain signal was converted into a time–frequency signal in order to achieve analysis from both the time domain and frequency domain perspectives and to initially solve the non-smooth and nonlinear problems of the signal. We then designed a novel bearing fault diagnosis model, which was based on the dilated-convolution-based residual model, and the MAB module and the global residual block were designed to increase the feature extraction ability of the model and improve its accuracy. To validate the noise immunity of the model for testing, the CWRU dataset was mixed with Gaussian white noise for the experiments, and the effectiveness of the proposed method was proven by comparative analysis with several advanced models Attention EfficientNet, RSG, and FDGRU and the classical network models Resnet18 and EfficientNet v2, respectively. Finally, the effectiveness of the designed MAB module and the GRB module was verified by ablation experiments.

From a theoretical perspective, the study advances the understanding of fault diagnosis in the presence of noise. By investigating the impact of noise on fault signals and proposing noise suppression and feature enhancement strategies, the study provides valuable insights into the challenges posed by noise in fault diagnosis. The study expands the knowledge base regarding the effects of noise on signal distortion, signal-to-noise ratio degradation, and increased risk of misdiagnosis. Additionally, the study contributes to the theoretical framework by proposing novel methods for noise suppression and feature enhancement, which can serve as a foundation for further studies in this field.

In terms of practical applications, the study findings offer practical solutions for improving fault diagnosis in real-world scenarios. By developing effective noise suppression techniques and feature enhancement strategies, the study provides practitioners with tools to enhance the accuracy and reliability of fault diagnosis systems. These findings have the potential to benefit various industries and domains that rely on fault diagnosis, such as manufacturing, transportation, and machinery maintenance. By addressing the challenges posed by noise, the research enables more precise and reliable identification of faults, leading to improved operational efficiency, reduced downtime, and cost savings.

Nevertheless, it is important to note that the methodology employed in this study did not address the potential influence of class imbalance on fault diagnosis. This limitation arises from the fact that the collected data predominantly consisted of healthy samples, with a smaller proportion of fault samples. As a result, the class imbalance issue was not explicitly considered in the current work. However, it is recognized that class imbalance can significantly impact the performance of fault diagnosis models, and addressing this concern will be a focus of future research endeavors. Subsequent investigations will strive to incorporate appropriate techniques or algorithms to mitigate the effects of class imbalance, thereby enhancing the robustness and generalizability of the fault diagnosis approach.

## Figures and Tables

**Table 1:** The configuration of model parameters.

Blocks	Layers	Kernel	Stride	Channel	Dilation Rate
-	Conv2d	5	2	64	-
-	MaxPool2d	3	2	-	-
BasicBlock 0	Conv2d	3	1	64	1
	Conv2d	3	1	64	1
BasicBlock 1	Conv2d	3	1	128	1
	Conv2d	3	1	128	1
BasicBlock 2	Conv2d	3	1	128	7
	Conv2d	3	1	128	7
BasicBlock 3	Conv2d	3	2	256	1
	Conv2d	3	2	256	1
Pool	AdaptiveAvgPool2d	-	-	-	-
FC	Linear(256,10)	-	-	-	-
Dropout	Dropout2d	-	-	-	-

**Table 2:** Description of the datasets.

Fault Diameter (Inches)	Bearing Conditions	Motor Load (HP)	Label
-	Normal	0/1/2/3	0
0.007	Inner fault	0/1/2/3	1
0.007	Ball fault	0/1/2/3	2
0.007	Outer fault	0/1/2/3	3
0.014	Inner fault	0/1/2/3	4
0.014	Ball fault	0/1/2/3	5
0.014	Outer fault	0/1/2/3	6
0.021	Inner fault	0/1/2/3	7
0.021	Ball fault	0/1/2/3	8
0.021	Outer fault	0/1/2/3	9

**Table 3:** The testing accuracy results of the MAB-DrNet and the six state-of-the-art methods under different SNR scenarios on the CWRU bearing dataset.

Method	SNR = −6 dB	SNR = −4 dB	SNR = −2 dB	SNR = 0 dB
LeNet5	79.28%	86.97%	92.15%	93.73%
ResNet 18	94.40%	96.99%	98.33%	99.33%
EfficientNet v2	94.40%	97.49%	98.83%	99.25%
Attention EfficientNet [37]	87.87%	89.86%	93.73%	95.91%
RSG [24]	-	96.67%	96.77%	98.32%
FDGRU [38]	-	94.86%	97.97%	99.19%
Ours	95.57%	97.91%	98.66%	99.33%

**Table 4:** The effect of the dilation rate on the model accuracy.

Model	d = 3	d = 5	d = 7	d = 9
ours	96.16%	97.24%	97.91%	97.08%

**Table 5:** The testing accuracy (%) results of DrNet, DrNet + GRB, DrNet + MAB, and DrNet + MAB + DrNet under different SNR scenarios on the CWRU bearing dataset.

Module	−6 dB	−4 dB	−3 dB	−2 dB	0 dB
DrNet (base)	94.15%	97.08%	97.91%	98.41%	98.83%
DrNet + GRB	95.49%	97.24%	98.16%	98.08%	99.00%
DrNet + MAB	94.32%	97.66%	97.99%	98.33%	99.33%
DrNet + MAB + DrNet	95.57%	97.91%	98.33%	98.66%	99.33%

## Data Availability

The data presented in this study are available upon request from the corresponding author. They are restricted to the experimental results.

## Nomenclature

DrNet	Dilated residual network
MAB	Max-average block
GRB	Global residual block
CNN	Convolutional neural network
STFT	Short-time Fourier transform
FFT	Fast Fourier transform
SNR	Signal-to-noise ratio
ReLU	Rectified linear unit
t-SNE	t-distributed stochastic neighbor embedding
HP	Horse Power
EDM	Electrical discharge machining
RSG	Residual building unit, soft thresholding, and global context
EDM	Electrical discharge machining
FDGRU	Gated recurrent unit for fault diagnosis
